# A multivariate Swedish national twin-sibling study in women of major depression, anxiety disorder, fibromyalgia, and irritable bowel syndrome

**DOI:** 10.1017/S0033291725000923

**Published:** 2025-04-28

**Authors:** Kenneth S. Kendler, Henrik Ohlsson, Michael Neale, Hanna van Loo, Judith G.M. Rosmalen, Jan Sundquist, Kristina Sundquist

**Affiliations:** 1 Virginia Institute for Psychiatric and Behavioral Genetics, Virginia Commonwealth University, Richmond, VA, USA; 2 Department of Psychiatry, Virginia Commonwealth University, Richmond VA, USA; 3 Center for Primary Health Care Research, Lund University, Malmö, Sweden; 4 Department of Psychiatry, University Medical Center Groningen, Groningen, Netherlands; 5 Department of Internal Medicine, University Medical Center Groningen, Groningen, Netherlands; 6 University Clinic Primary Care Skåne, Region Skåne, Malmö, Sweden; 7 Center for Community-Based Healthcare Research and Education (CoHRE), Department of Functional Pathology, School of Medicine, Shimane University, Japan

**Keywords:** Functional Somatic Disorders, Internalizing Psychiatric Disorders, Genetic Risk, Major Depression, Anxiety Disorders, Fibromyalgia, Irritable Bowel Syndrome

## Abstract

**Background:**

Functional Somatic Disorders (FSD) and Internalizing Psychiatric Disorders (IPD) are frequently comorbid and likely share familial/genetic risk factors.

**Methods:**

We performed a Common Factor Multivariate Analysis of 2 FSDs, Fibromyalgia (FM) and Irritable Bowel Syndrome (IBS), and two IPDs, Major Depression (MD) and Anxiety Disorders (AD), in five kinds of Swedish female–female relative pairs: monozygotic (n = 8,052) dizygotic (n = 7216), full siblings (n = 712,762), half-siblings reared together (n = 23,623), and half-siblings reared apart (n = 53,873). Model fitting was by full information maximum likelihood using OpenMx.

**Results:**

The best-fit model included genetic, shared environmental, and unique environmental factors. The common factor, ~50% heritable with a small shared environmental effect, loaded more strongly on the two IPDs (~0.80) than the 2 FSDs (0.40). Disorder-specific genetic effects were larger for the 2 FSDs (~0.30) than the 2 IPDs (~0.03). Estimated genetic correlations were high for MD and AD (+0.91), moderate between IBS and IPDs (+0.62), and intermediate between FM and MD (+0.54), FM and AD (+0.28), and FM and IBS (+0.38). Shared environmental influences on all disorders were present but small.

**Conclusions:**

In women, FSDs and IPDs shared a moderate proportion of their genetic risk factors, greater for IBS than for FM. However, the genetic sharing between IBS and FM was less than between MD and AD, suggesting that FSDs do not form a highly genetically coherent group of disorders. The shared environment made a modest contribution to the familial aggregation of FSDs and IPDs.

## Introduction

Functional Somatic Disorders (FSD) are characterized by clusters of somatic symptoms of unknown origin which, in the absence of detectable pathological abnormalities, are diagnosed solely by symptoms. Two of the best-studied FSDs are Fibromyalgia (FM) and Irritable Bowel Syndrome (IBS). These syndromes are relatively common (Nimnuan, Rabe-Hesketh, Wessely, & Hotopf, 2001) and, compared with chronic medical diseases with similar levels of symptoms, are associated with similar reductions in levels of functioning and quality of life (Joustra, Janssens, Bultmann, & Rosmalen, 2015). The clinical presentation of these syndromes is often marked by substantial comorbidity with internalizing psychiatric disorders (IPDs), in particular with Major Depression (MD) and Anxiety Disorders (AD) (Chang et al., 2015; Sibelli et al., 2016; Yepez et al., 2022; Zamani, Alizadeh‐Tabari, & Zamani, 2019).

Clarification of the genetic background of FSD could provide insight into etiological mechanisms as several studies suggest a familial/genetic predisposition to FM (Dutta et al., 2020; Magnusson, Turkiewicz, Rydén, & Englund, 2024; Markkula et al., 2009; Nielsen, Knudsen, & Steingrimsdottir, 2012) and IBS (Svedberg, Johansson, Wallander, & Pedersen, 2008). Which particular genes are associated remains unknown as genome-wide association studies of FSD are in early stages, with limited power and/or shallow phenotyping (Bonfiglio et al., 2018; Docampo et al., 2014; Schlauch et al., 2016). A recent large study identified six genetic susceptibility loci for IBS, of which four were associated with mood and anxiety disorders, expressed in the nervous system, or both (Eijsbouts et al., 2021).

Further insight into the nature of genetic vulnerabilities can be obtained by studying genetic liability shared with other disorders. In particular, consistent with the findings of clinical comorbidity, the literature also suggests an etiologic relationship between IPDs and FM and IBS. For example, we previously found, using family-genetic risk scores, substantially elevated genetic risks for MD and AD in cases of FM and IBS ascertained through the Swedish Medical registries (Kendler, Rosmalen, Ohlsson, Sundquist, & Sundquist, 2023a). A particularly relevant prior study utilized computer-assisted phone interview diagnosis in a subsample of the Swedish Twin Register. Kato et al performed a multivariate twin study suggesting genetic links between MD, Generalized Anxiety Disorder, IBS, and chronic widespread pain as a proxy for FM (Kato, Sullivan, Evengård, & Pedersen, 2009).

In this report, we seek to clarify further the genetic and environmental relationship between IPDs – represented here by MD and AD – and two of the more often studied FSDs: FM and IBS. We do so by applying traditional multivariate twin modeling methods to diagnoses of MD, AD, FM, and IBS obtained from Swedish medical registries. To maximize power, we use the traditional genetically informative sample of monozygotic (MZ) and dizygotic (DZ) twin pairs together with much larger samples of full siblings, half-siblings reared together, and half-siblings reared apart. These samples complement the information provided by traditional twin studies for both the effect of genetic factors (by the comparison of the degree of resemblance of full siblings to reared together half-siblings) and for the shared environment (by the comparison of reared together versus reared apart half-siblings).

In exploratory analyses, we fit a common factor model to these four disorders and are particularly interested in the degree to which the genetic influences for MD, AD, FM, and IBS derived from this common factor versus disorder-specific genetic effects. We will also examine the estimated genetic correlations among these four disorders. While we anticipate from multiple prior studies a high genetic correlation between MD and AD (Kendler et al., 1992; Roy et al., 1995), the genetic correlation between FM and IBS would be particularly informative about the degree to which two FSDs, which present with quite different symptom patterns, substantially share the same etiologic factors, including genetic vulnerabilities, as has been previously proposed (Wessely, Nimnuan, & Sharpe, 1999).

## Methods

We collected information on individuals from Swedish population-based registers with national coverage linking each person’s unique personal identification number which, to preserve confidentiality, was replaced with a serial number by Statistics Sweden. For the analysis, we double entered, from the Swedish Twin Registry, all female twin pairs with known zygosity and birth years between 1950 and 1990, and from the Swedish Multi-Generation Register all Swedish-born female full- and half-sibling pairs born between 1950 and 1990 within 5 years of each other. As detailed below, the rates of FM in males were too low to permit analysis. An individual could be included several times if she had several siblings or different types of siblings. Zygosity was assigned using standard self-report items, which, when validated against biological markers, were 95% to 99% accurate (Lichtenstein et al., 2002). Furthermore, using the Swedish national census and population registers, we assessed the cohabitation status for all pairs as the proportion of possible years they lived in the same household until the oldest turned 16. Among twins and full siblings, we only included pairs reared together for ≥90% of their possible years. For half-siblings, we included pairs reared together for ≥90% and pairs reared together ≤10% of the possible years, classifying these as pairs reared together or apart, respectively (see below for implications within the twin/family model). MD, AD, FM, and IBS were defined at the individual level using information on lifetime diagnoses from primary care data and the Swedish population-based registers which include hospital and out-patient specialist-based records (see Supplementary Material, Appendix Table 1). The variables were treated as binary, and the registration could occur at any time up till the end of the follow-up period on 2018-12-31.

We fitted a Common Factor model that aims to divide the sources of individual differences in liability to MD, AD, FM, and IBS into additive genetic (A), shared environment (C), and unique environment (E). The model assumes that MZ twins share 100% of their genes; DZ twins and full siblings share, on average, 50% of their genes; while half-siblings share, on average, 25% of their genes. The model also assumes that the shared environment (C), which reflects family and community experiences, is equal between MZ twins, DZ twins, and full siblings, while for half siblings C equaled 1 for pairs reared together and 0 for pairs reared apart. Finally, the unique environment (E) reflects experiences not shared by twins/siblings, random developmental effects, and measurement errors.

**Figure 1.**
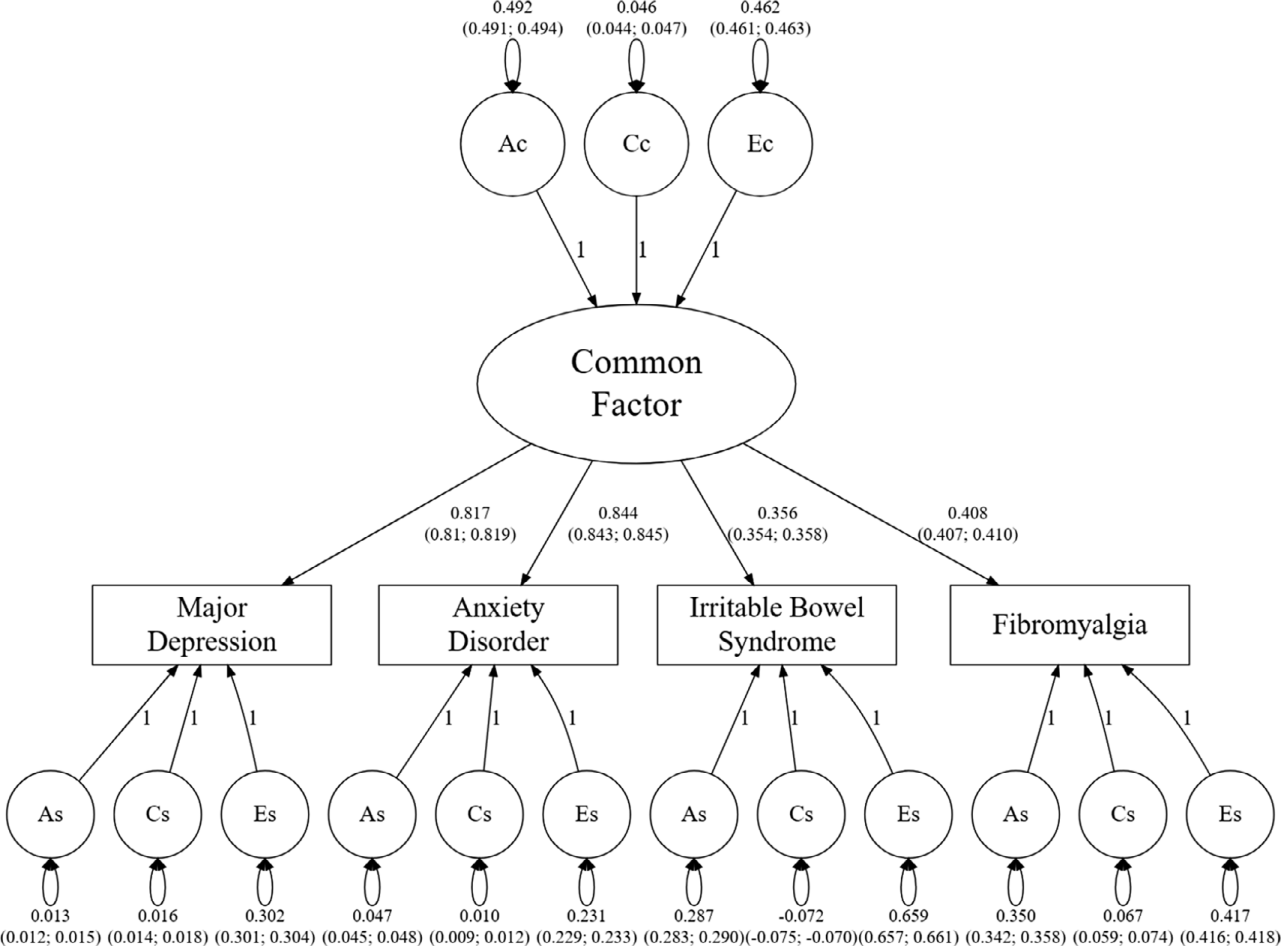
Parameter estimates (and 95% CIs) of a common factor model fitted to the twin-sibling data on Major Depression, Anxiety Disorder, Irritable Bowel Syndrome, and Fibromyalgia. A, C, and E stand for, respectively, additive genetic, shared (common) environment, and individual-specific environment, respectively. The subscript ‘c’ stands for ‘common’ – that it has a causal effect on the common factor – and the subscript ‘s’ for ‘specific’, mean that its effect was only on one of the specific disorders. The paths from the Common Factor to the four disorders represent standardized regression paths. The common and disorder-specific A, C, and E components were modeled as variances and therefore are depicted as double-headed variance paths specific to each component.

In the common factor model, the common factor (see Figure 1) is caused by additive genetic (A), common (C), and specific (E) environmental factors. The four individual phenotypes MD, AD, FM, and IBS are all caused by this latent factor. The residual variance of the four disorders is also partitioned into additive genetic, common, and specific environment components (As1–As4, Cs1–Cs4, and Es1–Es4). The variance components of the latent variable sum to 1 (the variance of the latent common factor), while the residual variance components sum to the residual phenotypic variation. We also tested for a twin-specific variance component (extra resemblance for twins above and beyond that seen for siblings) to the latent factor and the residuals. It did not markedly improve fit indicating no greater covariance for twins than sibs. Therefore, we decided on the model without these extra components. Following Verhulst et al. (2019), the model specifies variance components instead of path coefficients to avoid statistical problems when parameters have a lower bound of zero. Models were fit using the OpenMx R package (Neale et al., 2016). We secured ethical approval for this study from the Regional Ethical Review Board in Lund and no participant consent was required (No. 2008/409 and later amendments).

## Results

### Descriptive statistics

The widely varying sample sizes of our five kinds of relative pairs are seen in Table 1, ranging from 8,052 MZ pairs to 712,762 full sibling pairs. In our pairs from intact families, the lifetime prevalences of MD and AD were ~20%, IBS ~6%, and FM ~1% and moderately higher in our samples from half-sibling families.

**Table 1.** Sample size and within disorder cross-relative, within-individual cross-disorder and cross-disorder cross-relative tetrachoric correlations (with standard errors) for major depression, anxiety disorders, irritable bowel syndrome and fibromyalgia in our twin, full sibling and half sibling samples

Nature of correlations	Females	MZ twins (SHH)	DZ twins (SHH)	FS (0–5 years SHH)	HS (SHH)	HS (NoHH)
	N pairs	8,052	7,216	712,762	23,623	53,873
	% MD	19.6%	18.8%	20.8%	28.9%	26.8%
	% AD	18.6%	17.5%	19.4%	27.2%	25.1%
	% IBS	5.8%	5.1%	6.0%	7.0%	6.4%
	%FM	1.0%	0.9%	1.2%	2.3%	2.6%
Tetrachoric correlations (with standard errors)						
Within-disorder cross relatives	S1 MD × S2 MD	0.46 (0.02)	0.23 (0.02)	0.21 (0.00)	0.14 (0.01)	0.07 (0.01)
	S1 AD × S2 AD	0.45 (0.02)	0.22 (0.02)	0.24 (0.00)	0.17 (0.01)	0.10 (0.01)
	S1 IBS × S2 IBS	0.39 (0.03)	0.06 (0.05)	0.11 (0.00)	0.06 (0.02)	0.08 (0.02)
	S1 FM × S2 FM	0.63 (0.05)	–	0.29 (0.01)	0.24 (0.04)	0.10 (0.03)
Cross-disorder within-relative	S1 MD × S1 AD	0.69 (0.01)	0.68 (0.02)	0.69 (0.00)	0.69 (0.01)	0.69 (0.00)
	S1 MD × S1 IBS	0.30 (0.03)	0.23 (0.03)	0.27 (0.00)	0.24 (0.02)	0.27 (0.01)
	S1 MD × S1 FM	0.36 (0.05)	0.31 (0.06)	0.34 (0.00)	0.32 (0.02)	0.36 (0.01)
	S1 AD × S1 IBS	0.33 (0.03)	0.31 (0.03)	0.30 (0.00)	0.26 (0.02)	0.30 (0.01)
	S1 AD × S1 FM	0.29 (0.05)	0.37 (0.05)	0.31 (0.00)	0.28 (0.02)	0.30 (0.01)
	S1 IBS × S1 FM	0.31 (0.06)	0.25 (0.07)	0.29 (0.01)	0.28 (0.03)	0.26 (0.02)
Cross-disorder cross- relative	S1 MD × S2 AD	0.41 (0.02)	0.19 (0.02)	0.20 (0.00)	0.14 (0.01)	0.08 (0.01)
	S1 MD × S2 IBS	0.24 (0.03)	0.14 (0.03)	0.08 (0.00)	0.03 (0.02)	0.03 (0.01)
	S1 MD × S2 FM	0.34 (0.05)	0.06 (0.06)	0.10 (0.01)	0.04 (0.02)	0.03 (0.02)
	S1 AD × S2 IBS	0.23 (0.03)	0.10 (0.03)	0.10 (0.00)	0.07 (0.02)	0.03 (0.01)
	S1 AD × S2 FM	0.24 (0.05)	0.02 (0.07)	0.08 (0.01)	0.06 (0.02)	0.01 (0.02)
	S1 IBS × S2 FM	0.17 (0.07)	–	0.08 (0.01)	0.06 (0.03)	–

*Note:* –, Cannot be estimated; SHH, raised in the same household; NoHH, not raised in the same household. MD, major depression; AD, anxiety disorders; IBS, irritable bowel syndrome; FM, fibromyalgia.

Correlations for our four disorders within MZ pairs ranged from +0.39 for IBS to +0.63 for FM. Correlations were considerably lower and similar in DZ pairs and full siblings (except in FM where the DZ correlation was undefined as we had no concordant affected DZ pairs and IBS where the correlation was nearly twice as high in full siblings as DZ twins). This pattern of greater phenotypic correlation among MZ than DZ pairs suggests an important role of genetic liability for our disorders and similar results are suggested by a consistently higher correlation in full siblings versus half-siblings reared together. Evidence for a role for shared environment, however, is also present as the correlation for half siblings reared together was higher than those for half-siblings reared apart for MD, AD, and FM.

The within-person cross-disorder correlations were reassuringly similar across all relative groups equaling around +0.69 for MD and AD with nearly all other correlations ranging from +0.25 to +0.35. The cross-twin cross-disorder correlations were, as expected, more variable but consistently highest for MD-AD.

### Model fitting

We began with a full common factor model and fitted only a single alternative model that constrained all the C parameters to zero. This model fitted considerably worse by AIC than the full model (3,902,765 vs. 3,902,741), and so we focus here on the full model with parameter estimates and CIs presented in Figure 1.

The model has two notable features. First, the common factor is moderately heritable (a^2^ = 0.49) with a small contribution of shared environment (c^2^ = 0.04) and the remaining variance because of unique environmental effects (e^2^ = 0.47). The paths from the common factor were easily divisible into two groups with those to the IPDs being about twice as strong (ranging from +0.82 to +0.84) as those to the FSD (ranging from 0.36 to 0.41). Second, with respect to the residual disorder-specific factors, the results for MD and AD were similar. Both showed quite modest genetic-specific effects (accounting for ≤5% of disorder variance) and even smaller disorder-specific shared environments effects (accounting for ≤2% of disorder variance). By contrast, the disorder-specific unique environmental effects were much more substantial (~23% for ADs and 30% for MD). However, the disorder-specific effects differed moderately between our two FSDs. Disorder-specific genetic and shared environmental effects were both stronger for FM than for IBS and the opposite was seen for disorder-specific unique environmental effects.

The total genetic, shared, and individual-specific effects for our 4 disorders and their decomposition into common and disorder-specific effects disorders are seen in Table 2. In terms of total effects, IBS has by far the lowest estimated heritability (21%), with the heritabilities of MD, AD, and FM all estimated to be between 34% and 43%. Shared environmental effects were all quite modest ranging from 1% to 8%. Unique environmental effects were in a similar range for MD, AD, and FM (49%–61%) but were appreciably higher for IBS at 72%. This table depicts one anomaly – negative variance estimates for the shared environment for IBS. This result accords with the data, which reveal a DZ correlation of less than half of the MZ correlation, consistent with non-additive genetic variance or genotype by environment interaction for IBS. As usual for a classical twin study, non-additive and common environmental sources are confounded. For IBS the preponderance of effect appears non-additive, whereas for the other disorders, there is greater evidence of common environmental variation.

**Table 2.** Variance components (with 95% CIs), from the full common factor model

	Variance components				
	Total	Common	Specific	%Common	%Specific
a_MD_	0.34 (0.34; 0.35)	0.33 (0.32; 0.33)	0.01 (0.01; 0.02)	96%	4%
c_MD_	0.05 (0.04; 0.05)	0.03 (0.03; 0.04)	0.02 (0.01; 0.02)	66%	34%
e_MD_	0.61 (0.60; 0.62)	0.31 (0.30; 0.31)	0.30 (0.30; 0.30)	51%	49%
a_AD_	0.40 (0.40; 0.41)	0.35 (0.35; 0.35)	0.05 (0.05; 0.05)	88%	12%
c_AD_	0.04 (0.04; 0.05)	0.03 (0.03; 0.03)	0.01 (0.01; 0.01)	76%	24%
e_AD_	0.56 (0.55; 0.57)	0.33 (0.33; 0.33)	0.23 (0.23; 0.23)	59%	41%
a_IBS_	0.35 (0.30; 0.40)	0.06 (0.01; 0.11)	0.29 (0.28; 0.29)	18%	82%
c_IBS_	−0.07 (−0.13; 0.07)	0.01 (−0.04; 0.06)	−0.07 (−0.07; −0.08)	*	*
e_IBS_	0.72 (0.67; 0.77)	0.06 (0.01; 0.11)	0.66 (0.66; 0.66)	8%	92%
a_FM_	0.43 (0.39; 0.47)	0.08 (0.05; 0.11)	0.35 (0.34; 0.36)	19%	81%
c_FM_	0.07 (0.04; 0.11)	0.01 (−0.02; 0.04)	0.07 (0.06; 0.07)	10%	90%
e_FM_	0.49 (0.46; 0.52)	0.08 (0.05; 0.11)	0.42 (0.42; 0.42)	16%	84%

*Note:* MD, major depression; AD, anxiety disorders; IBS, irritable bowel syndrome; FM, fibromyalgia. A, additive genetic effect; C, common (or shared) environmental effects; E, individual-specific environmental effects. *% cannot be calculated because of the negative variance estimate.

When decomposing these effects, the pattern is similar for MD and AD with 90%–95% of their genetic risk, 65%–75% of their shared environmental effects, and 50%–60% of their unique environmental risk coming from the common factor. For IBS and FM, only 20%–30% of their genetic risk derives from the common factor. The total shared environmental effect for IBS is so small as to make decomposition of little value. For FM, the shared environmental effects are modest and largely disorder-specific.

Finally, the estimated genetic correlations among our four disorders are seen in Table 3. As expected, the correlation was very high for MD and AD (+0.91). The genetic correlations of our two IPDs and IBS were identical (+0.62) while the genetic correlation between FM and MD (+0.54) was considerably larger than that between FM and AD (+0.28). The genetic correlation between IBS and FM was surprisingly modest at +0.38.

**Table 3.** Genetic correlations estimated in bivariate models

	MD	AD	IBS	FM
MD	1	0.91 (0.85; 1.00)	0.62 (0.51; 0.75)	0.54 (0.38; 1.00)
AD		1	0.62 (0.53; 0.72)	0.28 (0.27; 0.44)
IBS			1	0.38 (0.23; 0.52)
FM				1

## Discussion

We applied, in this article, common factor multivariate structural equation modeling to a population-based sample of Swedish pairs of female–female half-siblings, full-siblings, and dizygotic and monozygotic twins, to clarify the degree of sharing of genetic and environmental risk factors between two Functional Somatic Disorders (FSD) – FM and IBS – and two Internalizing Psychiatric Disorders (IPD) – MD and AD. We would emphasize six results which we review in turn.

First, the identified common factor for these four disorders had a relatively precisely known heritability equal to almost exactly 50% with a much smaller contribution from the shared environment of slightly under 5%. As noted above, this heritability estimate derives not only from the traditional MZ-DZ comparisons but also from our relatively large samples of full- and half-siblings.

Second, the four loadings on this common factor were easily sorted into two groups with those for the two IPDs being relatively similar and roughly twice as strong as the two similar loadings for our FSDs, that is around +0.80 versus +0.40, respectively. Given the strength of these IPD loadings, it would be appropriate to consider this common factor as largely reflecting the liability to internalizing psychiatric disorders. If correct, this means that the common factor paths to FM and IBS can be assumed, at least as a first approximation, to represent the contribution of genetic risk for IPDs to those two FSDs.

Third, the disorder-specific contributions of genetic effects estimated in our model are also quite informative. The fact that the genetic-specific effects for MD and AD were quite small – 1% and 5% respectively – supports our contention that the general factor is really capturing nearly all the genetic effects for MD and AD or more broadly IPDs. The confirmation of this hypothesis – that our common factor largely reflects liability to the IPDs – means that, at a first approximation, we can divide the genetic risks for FM and IBS into two components – the proportion from the common factor which represents shared risk with IPDs and the unique effects which index genetic risks for the IPDs which are **not** shared with the IPDs. As seen in Table 2, we can estimate directly from our model the proportion of genetic effects for FM and IBS that are disorder-specific and these estimates are similar, and equal, respectively, 82% and 81%. While we clearly show a significant genetic relationship between the IPDs and FSDs, when examined as the proportion of the genetic variance that is shared versus unique, our answer is that only about 20% of the genetic risks for FM and IBS come directly from the common factor which in turn is dominated by the liability to IPDs.

Fourth, we were not able to include chronic fatigue syndrome in our analyses because of its rarity in the Swedish medical registries. Its inclusion would have given us the statistical possibility of identifying two common factors, one of which might have been largely for IPDs and the second for FSDs. However, we do have a way to estimate the viability of that two-factor model. The viability of the hypothesis that MD and AD share a common pool of genetic risk variants that would likely be important for other IPDs is strongly supported by the very high genetic correlation estimated between them: +0.91. However, the genetic correlation between FM and IBS was quite modest (+0.38) and argues against the existence of a robust common genetic factor underlying these two FSDs. This low genetic correlation also challenges prior hypotheses that the major FSDs are merely different names for the same disorder, caused by the tendency of specialists to focus on only those symptoms characteristic of their specialty, overlooking the communalities between patients with different FSD (Wessely et al., 1999). A further challenge to the genetic coherence of an IBS genetic common factor is that FM is substantially more highly corrected with MD (+0.54) than it is with IDB (+0.38).

Fifth, the estimate of heritability for our two IPDs – 34% for MD and 40% for AD – were well within the range of previous reports (Hettema, Neale, & Kendler, 2001; Hettema, Prescott, & Kendler, 2001; Kendler, Gatz, Gardner, & Pedersen, 2006; Sullivan, Neale, & Kendler, 2000). For IBS, our estimated heritability 0.35 (0.30–0.40) is congruent with prior estimates, often based on modest sample sizes which ranged from 0.22 to 0.57 (Bengtson, Rønning, Vatn, & Harris, 2006; Lembo, Zaman, Jones, & Talley, 2007; Morris-Yates et al., 1998; Svedberg et al., 2008) (with one negative study reporting no difference in MZ and DZ concordance rates (Mohammed et al., 2005)). We estimated the heritability of FM to equal 0.43 (0.39–0.47). Twin studies on FM reported heritabilities of 0.22 based on ICD-10 codes for myalgia and FM (Magnusson et al., 2024) and 0.51 with a case definition of FM based on questionnaire items (Markkula et al., 2009). Nielsen’s review of twin chronic widespread pain (Nielsen et al., 2012) indicated a heritability of around 50%, quite close to our estimate for FM. Our results are thus broadly consistent with this prior literature.

Sixth, while the genetic correlations of IBS with MD and AD were identical (r = +0.62), a different pattern was seen for FM. With FM, the genetic correlation with MD (+0.54) was appreciably higher than with AD (+0.28). These differences need to be followed up and given the low prevalence of FM in our sample, the 95% confidence intervals for those two genetic correlations overlap and could be a chance effect.

Previous studies explored familial co-aggregation of multiple FSDs and ISDs; both found co-aggregation between FM and MDD while results regarding IBS and AD were variable (Allen-Brady, Fyer, & Weissman, 2023; Hudson & Arnold, 2004). We previously found, using family-genetic risk scores, substantially elevated genetic risks for ID in cases of FM and IBS ascertained through the Swedish Medical registries, with associations with FM being stronger than those with IBS (Kendler et al., 2023a). In the current study, we found that the genetic correlations of our two IDs are higher for IBS than for FM.

A large study using interviews to assess ISD and FSD diagnoses in 31,318 twins using the Swedish Twin Register identified genetic links between MD, Generalized Anxiety Disorder, and FSD, particularly IBS, chronic widespread pain as a proxy for FM, chronic impairing fatigue, and recurrent headache (Kato et al., 2009). With six variables, a two-factor common pathway model was identified (which was not the case in our study with only 4 variables) and this was the best-fit model. The first latent factor was shared between IPD and FSD, interpreted as an affective factor, and the second one was shared with only the FSD. The affective common factor was more influenced by genetic liability and the second FSD factor by environmental factors. Our findings are broadly congruent with these results, despite the differences in a sample (both sexes vs. only female), assessment of diagnostic state (interview vs. medical registry), and inclusion of disorders. Our common factor, providing over 90% of the genetic risk for IPDs, while this is less than 20% for IBS and FM, resembled the affective factor in the previous study, while their second factor is reflected, in our model, by the disorder-specific effects that are more relevant for the FSDs than in IPDs.

### Limitations

These results should be considered in the context of two potentially important methodological limitations. First, case identification for all of our disorders is derived from physician diagnoses recorded in Swedish Medical registers. This might introduce some bias in our results, such as sampling more severe help-seeking cases than might be ascertained through a population survey. In addition, only a minority of those qualifying for an FSD diagnosis will receive one, and the chances of receiving a physician diagnosis are associated with personal characteristics such as sex (Tattan et al., 2024; Warren & Clauw, 2012). However, there are likely also some advantages of this registry-based approach since it is likely that patients have undergone a medical evaluation. It is interesting to note that in prior analyses using Swedish data (Kendler et al., 2023a), we found that for our two FSDs, 67% of cases of IBS and 60% of cases of FM were only diagnosed through primary care contacts.

Second, our results were limited to one sex – females. This was not by choice. The prevalence rates for FM in males across our twin sibling groups were too low (0.1%–0.2%) for meaningful analyses, as several of the key groups of twins and siblings lacked any concordant twin or sibling pairs. This prevented us from addressing the interesting question of the similarities of differences in the sharing of genetic and environmental risk factors for FSDs and IPDs, and across sexes.

## Conclusions

Using female–female pairs of MZ and DZ twins, siblings, and half-siblings, we tested a multivariate common factor model incorporating two classical FSDs – FM and IBS – and two standard IPDs – MD and AD. The best-fit model included a shared environmental variation, although the effects of this source of familial resemblance were relatively small. The common factor was moderately heritable but had much stronger effects on the two IPDs than the two FSDs. By contrast, the disorder-specific genetic effects were greater for the FSDs than for the IPDs. Overall, our model suggested that the genetic relationship between FSDs and IPDs was modest-to-moderate, and somewhat stronger for IBS than for FM. Interestingly, the genetic sharing between IBS and FM was much less than between MD and AD, suggesting that the FSDs do not form as genetically coherent a group of disorders as the IPDs.

## Supporting information

Kendler et al. supplementary material Kendler et al. supplementary material

## Data Availability

Kristina Sundquist MD PhD had full access to all the data in the study and takes responsibility for the integrity of the data and the accuracy of the data analysis.

## Supplementary material

The supplementary material for this article can be found at http://doi.org/10.1017/S0033291725000923.
