# Supplementary material for: A multivariate Swedish national twin-sibling study in women of major depression, anxiety disorder, fibromyalgia, and irritable bowel syndrome
Source: Psychol Med. 2025 Apr 28;55:e125. doi: 10.1017/S0033291725000923 (PMC12094625; doi:10.1017/S0033291725000923)
Supplement: Kendler et al. supplementary material [file S0033291725000923sup001.docx]

**APPENDIX**

**Table 1 ICD Codes for Diagnoses**

|  | **Registers used** | **ICD-codes** |
| --- | --- | --- |
| Fibromyalgia (FM) | *National Patient Register*  *Primary Care Data* | ICD-10: M79.7, ICD9: 729B |
| Irritable Bowel Syndrome (IBS) |  | ICD-10: K58, ICD9: 564B |
| Major Depression (MD) |  | ICD-8: 296.2, 298.0, 300.4; ICD-9: 296.2, 296.4, 298.0, 300.4; ICD-10: F32, F33. |
| Anxiety Disorders |  | ICD-8: 300.0, 300.2 ; ICD-9: 300A, 300C; ICD-10: F40, F41 |
|  |  |  |

**Table 2 - Description of Registers**

*Multi-Generation Register*

The Multi-Generation Register is a register made up of persons who have been registered in Sweden at some time since 1961 and those who were born in 1932 or later. These are called index persons. The register contains connections between index persons and their biological parents. There are about 11 million index persons in the register. The Multi-Generation Register is a part of the register system for Total Population Register, where information comes from the National Tax Board. Every year, a new version of the register is created, including new index persons who immigrated or were born during the year. Information from the Multi-Generation Register may be disclosed for research and statistical purposes. For more information, see Statistics Sweden, Background Facts, Population and Welfare Statistics 2017:2, Multi-generation register 2016. A description of contents and quality

*The Swedish Twin Registry*

The Swedish Twin Registry, managed by Karolinska Institutet is the largest of its kind and has become an invaluable resource for medical research. The Registry was established in the 1960s and contains information about some 87 000 twin pairs for which zygosity is known, both mono- and dizygotic pairs. For more information see: <https://ki.se/en/research/research-infrastructure-and-environments/core-facilities-for-research/the-swedish-twin-registry> .

*National Patient Register*

In the 1960's the National Board of Health and Welfare started to collect information regarding in-patients at public hospitals, the National Patient Register (NPR). Initially it contained information about all patients treated in psychiatric care and approximately 16 percent of patients in somatic care. The register at that time covered six of the 26 county councils in Sweden. In 1984, the Ministry of Health and Welfare together with the Federation of County Councils decided a mandatory participation for all county councils. From 1987, NPR includes all in-patient care in Sweden. Since 2001, the register also covers outpatient doctor visits including day surgery and psychiatric care from both private and public caregivers. For more information, see https://www.socialstyrelsen.se/en/statistics-and-data/registers/register-information/the-national-patient-register/

*Primary Care Data*

*
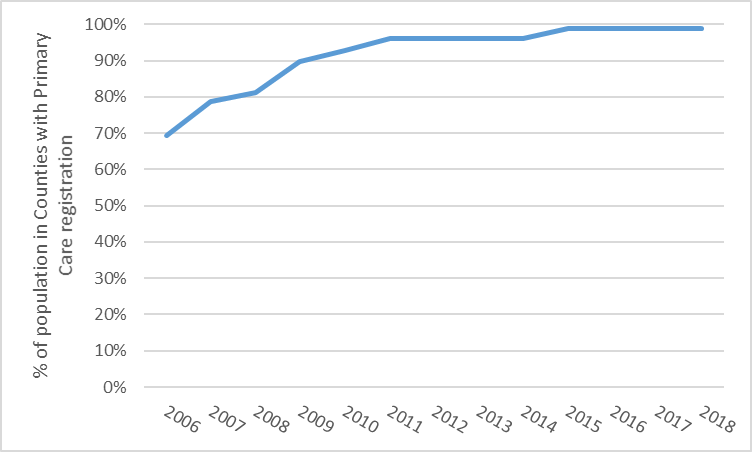
*We also used information from Primary Care. This is a research dataset including individual-level information on clinical diagnoses from primary health care centers. In the end of the follow-up period the registers cover almost 100% of the population. The figure shows the percentage of the entire Swedish population that resides in counties with primary care data. For more information see: Sundquist, J., Ohlsson, H., Sundquist, K., Kendler, KS. Common adult psychiatric disorders in Swedish primary care where most mental health patients are treated. BMC Psychiatry 17, 235 (2017). https://doi.org/10.1186/s12888-017-1381-4
